# A 2-D guinea pig lung proteome map

**DOI:** 10.1016/j.dib.2015.04.009

**Published:** 2015-05-27

**Authors:** Simone Schuller, Kjell Sergeant, Jenny Renaut, John. J. Callanan, Caitriona Scaife, Jarlath E. Nally

**Affiliations:** a University College Dublin, School of Veterinary Medicine, Belfield, Dublin 4, Ireland; b Luxembourg Institute of Science and Technology, Environmental Research and Innovation (ERIN) Department, 4422 Belvaux, Luxembourg; c Conway Institute for Biomolecular & Biomedical Research, Belfield, Dublin 4, Ireland; d Vetsuisse Faculty University of Bern, Länggassstrasse 128, 3012 Bern, Switzerland; e Ross University School of Veterinary Medicine, St. Kitts and Nevis, West Indies; f Bacterial Diseases of Livestock Research Unit, National Animal Disease Center, Agricultural Research Service, Department of Agriculture, Ames, IA, 50010, United States

## Abstract

Guinea pigs represent an important model for a number of infectious and non-infectious pulmonary diseases. The guinea pig genome has recently been sequenced to full coverage, opening up new research avenues using genomics, transcriptomics and proteomics techniques in this species. In order to further annotate the guinea pig genome and to facilitate future pulmonary proteomics in this species we constructed a 2-D guinea pig proteome map including 486 protein identifications and post translational modifications (PTMs). The map has been up-loaded to the UCD 2D-PAGE open access database (http://proteomics-portal.ucd.ie/). Transit peptides, N-terminal acetylations and other PTMs are available via Peptideatlas (ftp://PASS00619:NM455hi@ftp.peptideatlas.org/). This dataset is associated with a research article published in the Journal of Proteomics [1].

## Specifications table

**Table:** 

Subject area	Medicine, Infection biology
More specific subject area	Proteomic analysis of guinea pig lung
Type of data	• *Location of identified guinea pig lung proteins on a 12% acrylamide gel* • *Excel datasheet with identified proteins and corresponding peptide sequences from guinea pig lung* • *Mass spectrometry raw data and post translational modifications of seclected proteins from guinea pig lung*
How data was acquired	*2-D DIGE*, 5800 MALDI TOF/TOF (Applied Biosystems, Foster City, CA, USA)
Data format	2-D proteome map of lung (Progenesis Same Spots^®^), MS data of all identified proteins
Experimental factors	Guinea pig lung tissues were sonicated, proteins solubilized, labeled with cy dyes and analyzed via 2-D DIGE. Spots of interest were excised from a master gel and proteins identified via MS.
Experimental features	Guinea pig lung proteome map
Data source location	Dublin, Ireland
Data accessibility	The lung proteome map has been up-loaded to the UCD 2D –PAGE open access database (http://proteomics-portal.ucd.ie/). Transit peptides, N-terminal acetylations and other PTMs are available via Peptideatlas (ftp://PASS00619:NM455hi@ftp.peptideatlas.org/).

## Value of the data

• Description of the guinea pig lung proteome to facilitate future proteomic studies in this species.
• Aid in the annotation of the guinea pig genome.
• Description of PTMs on proteins related to infection.

## 1 Experimental design – materials and methods

### 1.1 Animal model and sample collection

Lung tissues from 12 weanling Hartley guinea pigs (Charles River Laboratories, UK) were used. Six animals were infected by intra-peritoneal injection of 5×10^2^ low passage in vitro cultivated *Leptospira interrogans* serovar Copenhageni (RJ 16441) in a final volume of 500 μl EMJH liquid culture medium. Six weight-matched control animals were injected with 500 μl of EMJH liquid culture medium only. Infected animals were sacrificed when moribund as described [1]. Lung tissues were collected, snap frozen in liquid nitrogen and stored at −80 °C until downstream analysis.

### 1.2 2-D DIGE, spot selection

2-DIGE was performed as described in [1]. A total of 1554 spots were successfully aligned across all 6 gels. A master gel was prepared loading 1000 µg of a mixture of equal amounts of proteins from infected and non-infected lung tissue. Proteins were separated in two dimensions, similarly to the method used for 2-D DIGE. Briefly, 24 cm Immobiline DryStrip IPG strips pH 4–7 (GE Healthcare, Buckinghamshire, UK) were rehydrated overnight at room temperature with 1000 μg of labeled proteins in rehydration solution (30 mM DTT, 0.5% IPG buffer, bromophenol blue, labeled sample and solubilization buffer containing 7 M urea, 2 M thiourea, 1% ASB-14). Isoelectric focusing was then performed using an Ettan™ IPGphor IEF System (GE Healthcare, Buckinghamshire, UK) at 3500 V for 75,000 VHrs (step 1), a gradient of 8000 V for 10 min (step 2), 8000 V for 1 h (step 3), and 100 V for 5 h (holding step). Strips were transferred into equilibration buffer (6 M urea, 75 mM Tris–HCl, pH 8.8, 29,3% glycerol, 2% SDS and 0.002% bromophenol blue) with added 1% DTT for 10 min, followed by incubation in equilibration buffer containing 2.5% iodoacetamide for 10 min. The strips were overlaid on 12% acrylamide gels. Agarose gel with bromophenol blue (tracking dye) was used to seal the strips and the gels run at 0.5 W/gel for 1 h and 2 W/gel overnight using a DaltSix electrophoresis unit (GE Healthcare, Buckinghamshire, UK). The power was increased the following morning to 17 W/gel until the tracking dye reached the bottom edge of the gel. The master gels were then stained with SyproRuby^®^stain, aligned with the DIGE gels and 533 spots selected for protein identification [1].

### 1.3 Protein digestion and identification.

Spots of interest were excised from stained master gels and digested with trypsin using the fully automated Ettan® Spot handling Workstation (GE Healthcare, Buckinghamshire, UK). After digestion, the peptides were solubilized and 0.7 μl of the peptide mixture spotted on a MALDI target plate. 1 μl of matrix solution (alpha cyano-4-hydroxycinnamic acid in 50% ACN/0.1% TFA) was added and the sample was allowed to dry at ambient laboratory conditions. All MS and MS/MS analyses were performed using a 5800 MALDI TOF/TOF (Applied Biosystems, Foster City, CA, USA), internally calibrated with the known masses of trypsin autocleavage products in MS and externally with fragments from Glu-fibrinopeptide in MS/MS. For each sample, one MS spectrum was acquired and the 8 most intense precursors were subsequently selected for MS/MS analysis. An Applied ProteinPilot platform (version 4.5, Biosystems) was used for database searches on an in-house MASCOT server (version 2.3, Matrix Science). Peaks with a signal to noise ratio of more than 10 for MS-analysis and more than 5 for MS/MS analysis were included in the peak list. A maximum of 250 peaks was allowed for each spectrum. Combined MS and 8 MS/MS spectra from each spot were used to perform a search against the guinea pig protein-database (downloaded from NCBI server on 17/11/2011, containing 22,245 sequences) and subsequently against the guinea pig EST-database (downloaded on the same day containing 119,850 entries), a rodent protein database (downloaded 04/04/2012 containing 316,675 sequences) and the NCBI bacteria database (Downloaded 04/04/2012 containing 27,505,724 sequences). A mass window of 100 ppm for the precursor and 0.75 Da for the fragments was tolerated. During the database searches, the following parameters were defined: two missed cleavages, fixed carbamidomethylation of cysteine, variable oxidation of methionine and tryptophan to kynurenine or double oxidation to N-formylkynurenine. Proteins were considered as being identified when two peptides matched with a score above 40, the peptide threshold score or when one high-scoring peptide together with the MS-data resulted in a protein expect value <01e-005. All identifications were manually validated, as previously described [2]. During the acquisition of data, an effort was made to explain as many as possible of the peaks observed in the MS spectra, resulting in an increase in the sequence coverage for the reported identifications, the identification of multiple proteins in an important number of spots, and also in the discovery of post translational modifications (PTMs). Semi-tryptic peptides (peptides with unexpected, non-tryptic cleavage sites), were predicted using SignalP or MitoProt (expasy.org/tools). More specific PTMs, such as the redox-sensitivity of the reported oxidized cysteine and the proteolytic processing, were confirmed through literature searches.

### 1.4 Creation of the guinea pig lung proteome map

**Fig. 1:**
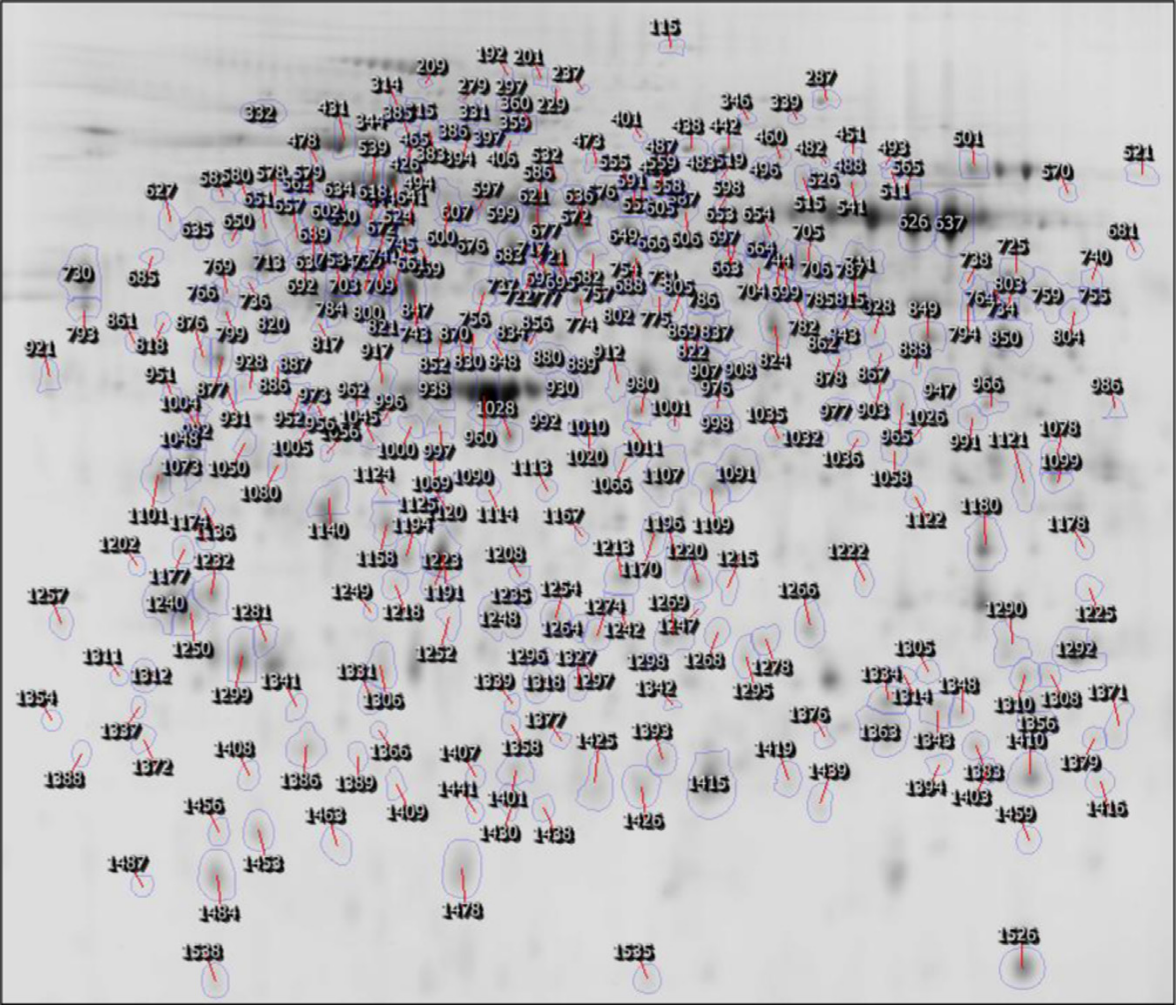
2-D guinea pig lung proteome map. Proteins from lung tissue of non-infected guinea pigs (n=6) and guinea pigs infected with 5x10^2^*Leptospira interrogans* serovar Copenhageni RJ 16441 (n=6) were separated on a 12% polyacrylamide gel using a pH 4–7 range IPG strip. The locations of all spots for which protein identifications were retrieved are shown. Spot numbers correlate with numbers in supplemental Table 1.

**Fig. 2:**
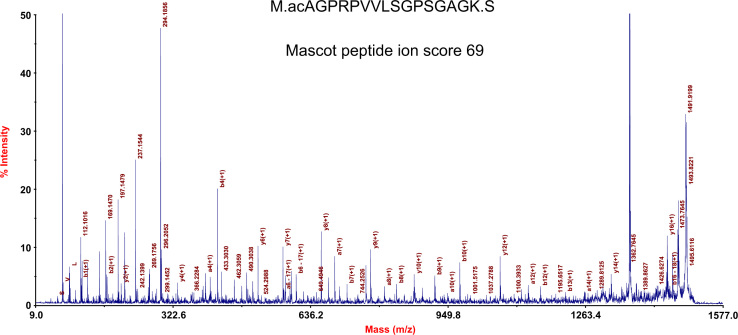
MS/MS spectra and identified peptide sequence of Guanylate kinase-like protein (spot 1403). Acetylation of the α-amino group of a residue C-terminal to methionine was observed, but the site does not correspond to the predicted start codon, indicating that the start codon might be mispredicted. BLAST-alignment indicates that most homologous mammalian proteins are indeed 21 amino acids shorter.

**Fig. 3:**
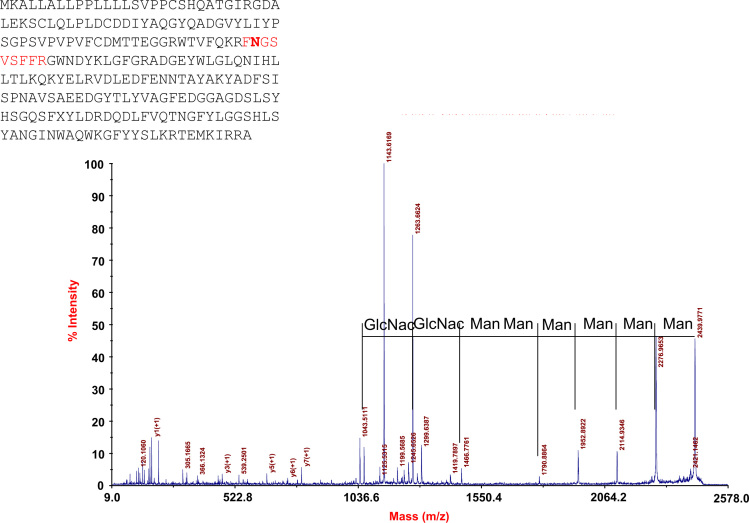
MS/MS spectrum of the glycosylated peptide identified in the spots 1158 and 1167. The protein identified in these spots is the microfibril-associated glycoprotein 4-like protein and the identified peptide sequence is indicated in red.

**Fig. 4:**
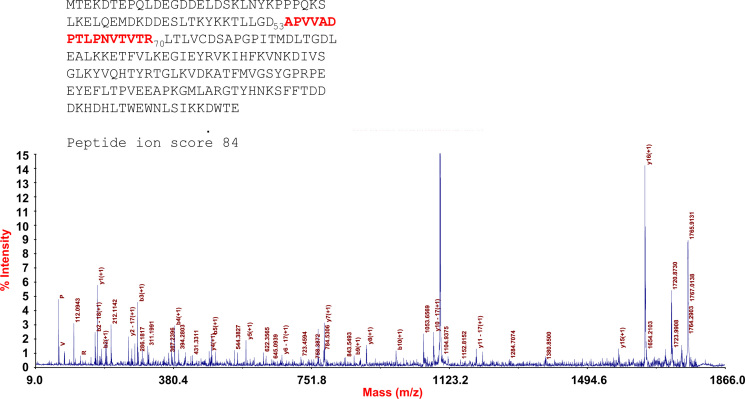
MS/MS spectrum of the semitryptic peptide identified as rho GDP-dissociation inhibitor 2-like protein in spot 1522. The peptide was cleaved between residue 52 and 56 consistent with caspatase-3-catalyzed cleavage, which renders the inhibitor unable to regulate Rho-like proteins.

All spots for which protein identifications had been found were selected and a proteome map constructed using the Progenesis Same Spots^®^ software (Fig. 1). This lung proteome map has been up-loaded to the UCD 2D-PAGE open access database (http://proteomics-portal.ucd.ie/). MS data for all identified proteins was summarized in Supplemental Table 1. Spectra corresponding to these identifications including those that allowed the identification of transit peptides, N-terminal acetylations and other PTMs are available via Peptideatlas (ftp://PASS00619:NM455hi@ftp.peptideatlas.org/). Spectra and sequences of two proteins with interesting PTMs and one semitryptic peptide are presented in Figs. 2–4.

## Supplementary Information

Table 1 Mass spectrometric data of all identified proteins. Spots are shown in ascending order of spot numbers. PMTs are indicated. Spot numbers correlate with numbers shown in Fig. 1.
